# From Broad-Spectrum Biocides to Quorum Sensing Disruptors and Mussel Repellents: Antifouling Profile of Alkyl Triphenylphosphonium Salts

**DOI:** 10.1371/journal.pone.0123652

**Published:** 2015-04-21

**Authors:** Alberto J. Martín-Rodríguez, Jose M. F. Babarro, Fernando Lahoz, Marta Sansón, Víctor S. Martín, Manuel Norte, José J. Fernández

**Affiliations:** 1 Institute for Bioorganic Chemistry “Antonio González”, Center for Biomedical Research of the Canary Islands (CIBICAN), Department of Organic Chemistry, University of La Laguna, Av. Astrofísico Francisco Sánchez 2, 38206 La Laguna, Tenerife, Spain; 2 Oceanic Platform of the Canary Islands (PLOCAN), Carretera de Taliarte s/n, 35214 Telde, Gran Canaria, Spain; 3 Department of Biotechnology and Aquaculture, Instituto de Investigaciones Marinas CSIC, Eduardo Cabello 6, 36208 Vigo, Spain; 4 Department of Fundamental & Experimental Physics, Faculty of Sciences, University of La Laguna, Av. Astrofísico Francisco Sánchez s/n, 38206 La Laguna, Tenerife, Spain; 5 Department of Botany, Ecology and Plant Physiology, Faculty of Sciences, University of La Laguna, Av. Astrofísico Francisco Sánchez s/n, 38206 La Laguna, Tenerife, Spain; University Hospital of the Albert-Ludwigs-University Freiburg, GERMANY

## Abstract

‘Onium’ compounds, including ammonium and phosphonium salts, have been employed as antiseptics and disinfectants. These cationic biocides have been incorporated into multiple materials, principally to avoid bacterial attachment. In this work, we selected 20 alkyl-triphenylphosphonium salts, differing mainly in the length and functionalization of their alkyl chains, in fulfilment of two main objectives: 1) to provide a comprehensive evaluation of the antifouling profile of these molecules with relevant marine fouling organisms; and 2) to shed new light on their potential applications, beyond their classic use as broad-spectrum biocides. In this regard, we demonstrate for the first time that these compounds are also able to act as non-toxic quorum sensing disruptors in two different bacterial models (*Chromobacterium violaceum* and *Vibrio harveyi*) as well as repellents in the mussel *Mytilus galloprovinciali*s. In addition, their inhibitory activity on a fouling-relevant enzymatic model (tyrosinase) is characterized. An analysis of the structure-activity relationships of these compounds for antifouling purposes is provided, which may result useful in the design of targeted antifouling solutions with these molecules. Altogether, the findings reported herein provide a different perspective on the biological activities of phosphonium compounds that is particularly focused on, but, as the reader will realize, is not limited to their use as antifouling agents.

## Introduction

**Fig 1:**
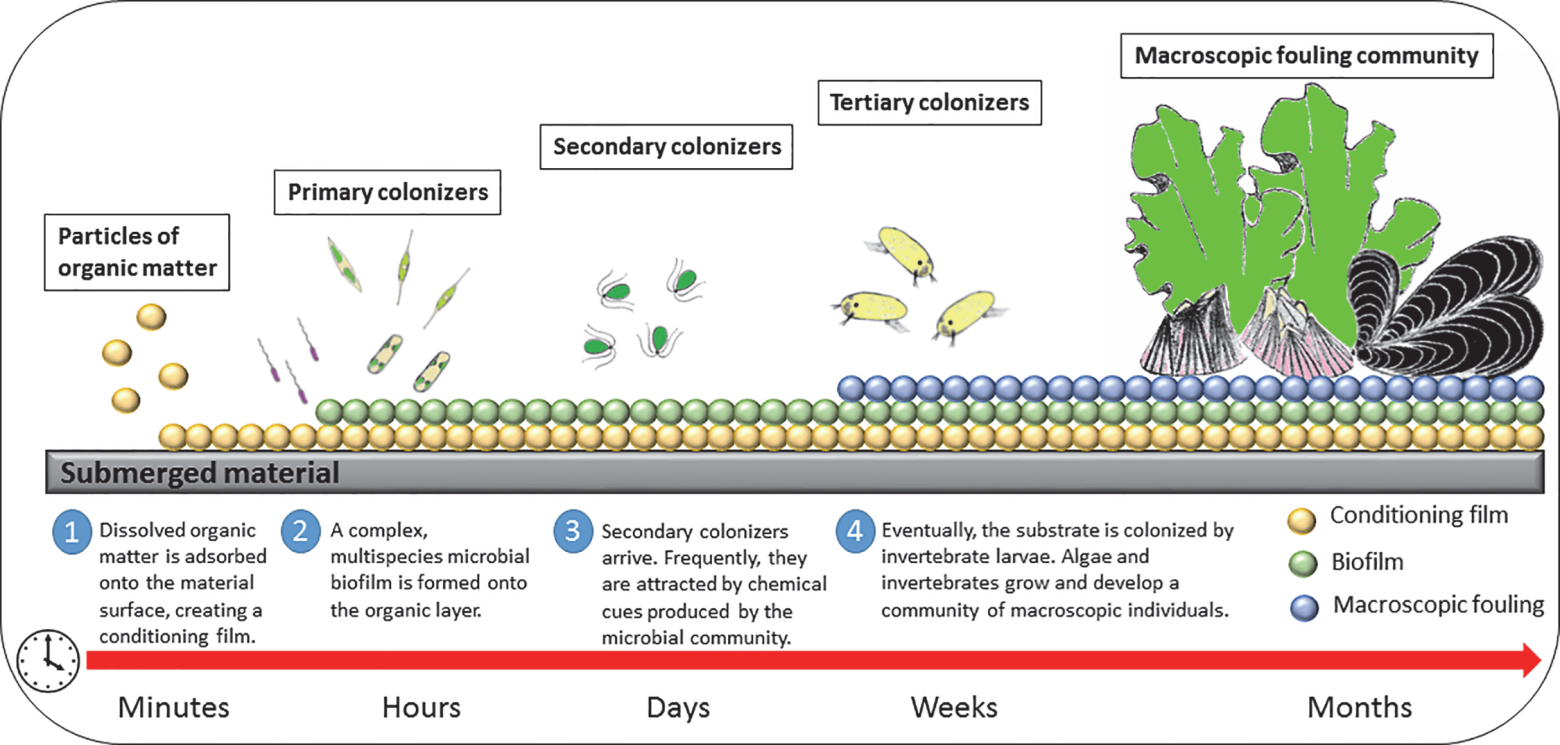
Schematic representation of the biofouling process.

Marine biofouling is a deleterious process that imposes a plethora of costly problems to human activities in the ocean, particularly to the shipping industry [1]. It is estimated that the costs associated to this phenomenon already exceed US $200 billion every year [2]. Biofouling is a complex phenomenon that involves a wide array of organisms, from microbes to invertebrates. It is often depicted as a successional process with four main stages, illustrated with an hypothetical material surface that is submerged in the sea (Fig 1): 1) adsorption, from the first seconds after immersion, of organic particles onto the submerged surface, with the development of a so-called ‘conditioning film’ that constitutes the molecular fouling and promotes the 2) arrival of primary colonizers, initially (first 24 h) pioneer motile bacteria and, within the first days, an array of microorganisms, with bacteria and benthic diatoms as the main representatives, that form complex multispecies biofilms (microfouling) and tend to promote the 3) settlement of macroalgal zoospores (e.g. ulvophycean) and 4) invertebrate larvae (e.g. mussel pediveligers, barnacle cyprids) that end up forming a complex macroscopic fouling community. Consequently, to characterize the antifouling profile of any given substance accurately, it is necessary to conduct bioassays with key organisms that are representative of the different stages of this phenomenon [3].

Over the second half of the past century, the problem had been relatively under control through the use of organotin compounds, in particular bis-(tris-*n*-butyltin)oxide (TBTO). However, increasing evidence of the detrimental environmental impact of these chemicals arose from the 1980s [4–6]. That increasing concern led to a stepwise prohibition of organotin compounds in antifouling paint formulations that was established by the International Maritime Organization and fully entered into force in September 2008.

Current antifouling research focus on the design of less harmful solutions that include: the tethering of biocides to the coating matrix, which remain fixed or are released to the surroundings in a controlled fashion (self-polishing antifouling systems) [1,7], low-surface-energy fouling-release coatings [8], hybrid antifouling-fouling release coatings [9], engineered microtopographies [10,11], enzyme-based coatings [12,13] or natural product antifoulants [14,15]. As biofouling begins with the formation of a biofilm, which in turn conditions the subsequent settlement of macroscopic organisms (Fig 1) [1,16], the use of inhibitors of bacterial cell-to-cell communication or quorum sensing (QS) has emerged as a non-toxic mechanism for the control of the early stages of the biofouling process [17].

Cationic compounds, such as quaternary ammonium and phosphonium salts, have been used since the 1930s as disinfectants and antiseptics and they have stood out due to their broad-spectrum antimicrobial activity and relatively low toxicity [18]. In comparison, phosphonium salts display better antimicrobial properties than their ammonium counterparts, either as single molecules in solution [19] or in their polymeric forms [20]. Phosphonium cations also exhibit higher thermal stabilities and ionic conductivities [21,22]. Their use has not been restricted to industrial environments and even in the biomedical field, phosphonium salts have been proved to display better antitumoral activities and lower toxicities than ammonium salts [23].

Over the last years, the abovementioned properties had led to the inclusion of phosphonium moieties in polymers for biomedical applications [22,24], water treatment [25], food packaging [26] or antifouling purposes [27,28]. They have been included in rubbers [29] or clays [30,31] to confer antimicrobial properties on these materials. As alkyl-triphenylphosphonium cations readily trespass the lipid bilayers, they have been employed as carriers to deliver drugs and therapeutics inside the mitochondria [32,33] or genes inside cells [34]. Other fields of application of phosphonium cations include the supramolecular chemistry [35,36], and the design of smart materials due to their self-assembly properties [37].

**Fig 2:**
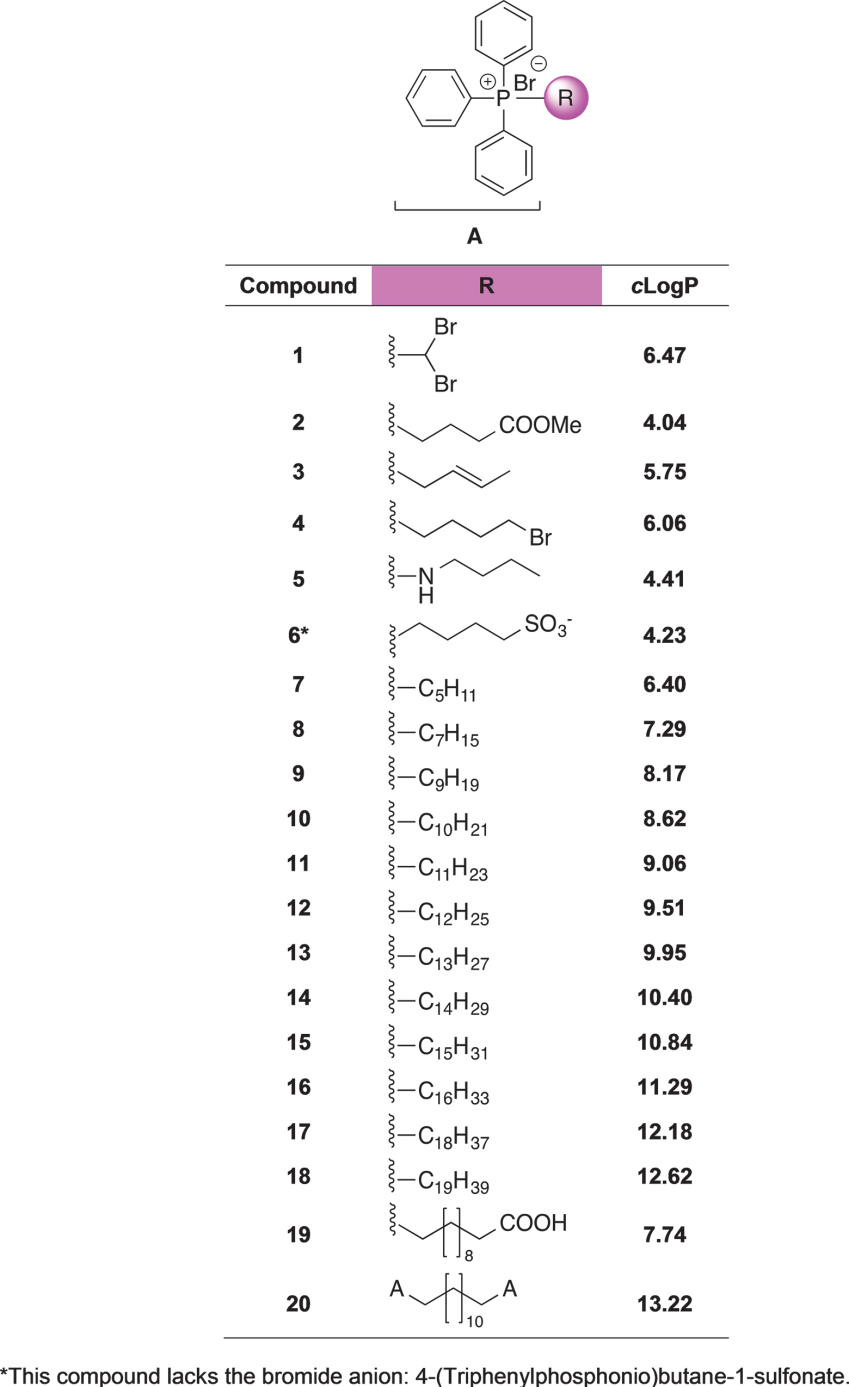
Structures and cLogP values of the compounds used in this study.

Despite such an intensive research, there was still a missing piece: a comprehensive assessment of their profile as antifouling agents with relevant fouling species that goes beyond the classical view of these compounds as broad-spectrum antimicrobials. To that end, 20 alkyl-triphenylphosphonium salts (Fig 2), differing in the length and functionalization of their alkyl chains, were selected for their study in a multidisciplinary approach that included their biological evaluation towards a wide panel of marine fouling organisms, an innovative use as bacterial QS disruptors and mussel repellents, and an enzymatic and fluorescence-based characterization as tyrosinase inhibitors, an enzyme that plays an essential role in mussel byssal production. Overall, the findings reported herein provide a detailed description, based on the chemical structure, of the profile of these molecules as active ingredients in antifouling coatings not necessarily as biocides but also as non-toxic repellents and disruptors of key processes for the biological colonization of immersed substrata.

## Materials and Methods

### Chemicals

A total of 20 triphenylphosphonium salts were used in this study (Fig 2). Compounds **1**–**3**, **11** and **15** were obtained from the chemical library of the Institute for Bioorganic Chemistry “Antonio González”, University of La Laguna. Compounds **4**–**7** were purchased from Sigma-Aldrich, and compounds **9**, **10**, **14** and **16** were acquired from Alfa-Aesar. Compounds **8**, **13**, and **17**–**20** were synthesized according to [38]. Briefly, a mixture of triphenylphosphine and the corresponding alkyl bromide was refluxed for 48 h. The resulting salt was filtered, washed in ether (x3) and dried. General methodological information and spectroscopic data relative to the synthetic products are available in S1 File.

Octanol/water partition coefficients were calculated with MarvinSketch 6.0.0 (ChemAxon Ltd., Budapest, Hungary). Unless otherwise stated, product samples were dissolved in dimethylsulfoxide (DMSO) at a concentration of 40 mM and conserved at -20ºC. TBTO (Sigma-Aldrich) was used as antifouling standard.

### General considerations for bioassays

All the biological assays were run by triplicate. Unless otherwise stated, the cut-off concentration was set at 100 μM and the maximum proportion of solvent was 0.25% (v/v). A product causing no effect at this concentration was considered not active. For the active products, serial dilutions were performed to calculate the half-maximal inhibitory concentrations (IC_50_) or the minimal inhibitory concentrations (MIC). Unless otherwise stated, the test concentrations were 100, 50, 25, 10, 5, 2.5, 1, 0.5 and 0.1 μM. Dose-response curves were adjusted with GraphPad Prism 5 software using a four-parameter non-linear regression model.

### Bioassays with marine bacteria

Six strains of marine bacteria were purchased from the Spanish Type Culture Collection (CECT): *Cobetia marina* CECT 4278, *Pseudoalteromonas atlantica* CECT 579, *Shewanella algae* CECT 5021, *Vibrio alginolyticus* CECT 521, *Vibrio anguillarum* CECT 4347 and *Cellulophaga lytica* CECT 5014. Growth inhibition was assessed in Marine Broth (Conda) according to CLSI guidelines for broth microdilution susceptibility testing [39]. The incubation time was 24 h and the incubation temperature was 30ºC. Flat-bottom microtiter plates were employed (Nunc 167008). Bacterial growth inhibition was determined by measuring the optical density at 625 nm (OD_625_).

### Bioassays with marine-derived fungi

Three strains of marine-derived fungi were kindly provided by Dr. Á. Trigos (University of Veracruz, Mexico). The strains were isolated from reef organisms collected from the Veracruz Reef System and identified as *Aspergillus* sp., *Fusarium* sp. and *Alternaria* sp. [40].

Fungal strains were cultured in Potato-Dextrose Agar (PDA) supplemented with filtered seawater (FSW) (50% v/v) at 26ºC for 5 days prior to experiments. Bioassays for fungal growth inhibition were conducted in 96-well plates in RPMI-1640 medium (+ *L*-Glutamine,—NaHCO_3_, Biochrom) supplemented with 2% glucose according to the EUCAST protocol [41]. Briefly, fungal inocula were prepared by addition of 5 ml of sterile saline solution to the cultures. Gently swabbing released conidia. Inocula were adjusted to 2–5 x 10^6^ cfu ml^-1^ by counting in a haemocytometer and diluted 1:10 in RPMI-1640 medium before dispensing into the wells (100 μl) in the presence of the appropriate dilution of the test products in RPMI (100 μl). The plates were incubated for 5 days at 26ºC. The calculation of the half-maximal inhibitory concentrations (IC_50_) was performed using OD_405_ as endpoint [42].

### Bioassays with diatoms

Five strains of benthic diatoms, *Nitszchia* sp. BEA 0497, *Navicula* cf. *salinicola* BEA 0055, *Phaeodactylum tricornutum*, *Cylindrotheca* sp. and *Amphora* sp. were used to study the effect of the compounds on microalgal growth. The *Nitzschia* and *Navicula* strains were purchased from the Spanish Bank of Algae (Marine Biotechnology Center, University of Las Palmas de Gran Canaria). The other three strains were kindly provided by Dr. G. Courtois (University of Las Palmas de Gran Canaria). Diatoms were cultured at 19±1ºC in Erlenmeyer flaks (250 ml) containing 150 ml of Guillard’s F/2 medium, and subjected to a photoperiod of 18:6. Tests were run in 48-well plates. Inocula were prepared by adjusting diatom concentration to 2–4 × 10^6^ cells ml^-1^ using a Neubauer chamber. Test products were dissolved in F/2 medium (500 μl) to which diatom inocula (500 μl) were added. Thus, the final assay volume was 1 ml and the starting cell densities were ca. 1–2 x 10^6^ diatoms ml^-1^. Plates were incubated under the abovementioned conditions for 5 days and then chlorophyll-*a* (Chla) was quantified.

In order to extract Chla, the content of each well was transferred to a microcentrifuge tube and centrifuged at 10,000 rpm for 10 min. The supernatants were discarded and 200 μl of DMSO were added to the pellets. The tubes were incubated at 65ºC for 2 h in total darkness and vortexed every 30 min. Then, the content of each tube was transferred to a 96-well plate and the amount of Chla was determined spectrophotometrically [43]. Path length correction factor for the DMSO extracts was determined [44].

### Bioassays with macroalgal spores

To evaluate the effect of the phosphonium salts on the germination of macroalgal spores, *Gayralia oxysperma* (Kützing) K.L.Vinogradova was selected as model organism. *G*. *oxysperma* (Ulotrichales, Chlorophyta) is a cosmopolitan member of the Ulvophyceae [45]. Unlike *Ulva* species, which produce biflagellate male and female gametes, as well as quadriflagellate spores, *G*. *oxysperma* only produces biflagellate spores. This is an advantage as bioassays can be conducted straightforwardly without the need to distinguish between gamete-producing and zoospore-producing plants. *G*. *oxysperma* specimens were collected from the upper eulittoral at El Médano, Tenerife, Canary Islands (UTM 28R 348359 3102405). Voucher specimens are deposited as TFC Phyc 14912 (Herbarium University of La Laguna). Fresh fertile fragments were selected and placed in Petri dishes. Spores were then released in Von Stosch Solution (VSS) by the osmotic method [46,47]. Bioassays were conducted in flat-bottom 96-well plates as described by Chambers and co-workers [48], with slight modifications. Each well was filled with 50 μl of the appropriate dilution of the products in VSS to which 50 μl of spore inoculum (ca. 2 x 10^5^ spores ml^-1^) were added. Plates were incubated at 19±1ºC for 6 days. After the incubation time, the bottom of each well was examined for the presence of germinated spores with an inverted microscope. A spore was considered as germinated when the germ tube was visible. The MIC was recorded as the lowest concentration inhibiting spore germination.

### Artemia salina tests

*Artemia salina* cysts (INVE Aquaculture, Ghent, Belgium) were hatched in brackish water (30 ‰ salinity) at 28ºC with aeration and under constant light. Newly hatched instar I nauplii were harvested for bioassays. Tests were conducted in 96-well plates (15±5 nauplii per well, test volume = 200 μl). The number of dead and alive individuals was recorded after 24 h of incubation at 28ºC and 24-hour photoperiod.

### Quorum sensing bioassays with Chromobacterium violaceum

The reporter strain *C*. *violaceum* CVO26 (CECT 5999) was used to screen the ability of the products to interfere with violacein production, a QS-regulated phenotype. *C*. *violaceum* CVO26 is a mini-Tn5 mutant that depends on an exogenous source of autoinducer (AI) (*N*-hexanoyl homoserine lactone, HHL) for violacein production. *C*. *violaceum* CVO26 was cultured in LB broth (Sigma-Aldrich) supplemented with 25 μg ml^-1^ kanamycin. Inocula were prepared by dilution (1:100) of an overnight culture of the reporter strain [49]. One hundred microliters of inoculum with HHL (Sigma-Aldrich, 6 μM) or without the AI were added to 96-well plates containing 100 μl of the appropriate dilutions of the test products in LB. Thus, two sets of plates were prepared. One set of plates (without HHL) was used to evaluate the effect of the products on bacterial growth, whereas the other set of plates (with HHL) was used to evaluate their effect on violacein production. Both sets of plates were incubated at 30ºC with agitation (150 rpm) for 18 h.

Growth inhibition was quantified in the first batch of plates by re-suspending bacterial pellets and measuring the OD_625_. From the second batch of plates violacein was extracted and quantified [50]. Briefly, the plates were dried overnight (60ºC) and violacein was re-solubilized by the addition of 200 μl of DMSO. The plates were shaken for 3 h and then the OD_590_ was determined.

### Quorum sensing bioassays with Vibrio harveyi

*V*. *harveyi* BB120 (wild type strain), BB170 (luxN::Tn5kan), BB886 (luxPQ::Tn5kan) and BB721 (luxO::Tn5lacZ) were acquired from ATCC. To screen the ability of the compounds to interfere with QS, the test products were serially diluted in 100 μl of Autoinducer Bioassay (AB) medium [51] using white, clear-bottom 96-well microtiter plates (Costar 3610) as assay platform. Aerobic bacterial cultures were incubated overnight (30ºC) and diluted 1:50. One-hundred μl of the diluted cultures were dispensed inside each well in the microtiter plate. That gave a starting cell density of 1–2 x 10^7^ cfu ml^-1^. The plates were covered with a sterile sealing film. Luminescence and OD_600_ were monitored every 15 min over 18 h with a multimode plate reader (Perkin-Elmer EnSpire) in order to correlate the effects of the products on both the growth and bioluminescence kinetics.

### Tyrosinase inhibition assays

Mushroom tyrosinase (EC 1.14.18.1) was purchased from Sigma-Aldrich. Tyrosinase inhibition assays were conducted as described in [52] with slight modifications. First, in order to characterize the Michaelis-Menten parameters of the enzyme kinetics, 100 μl of a mushroom tyrosinase solution (25 U) in sodium phosphate buffer (50 mM, pH 6.5) were pipetted in each well of a flat-bottom microtiter plate (Nunc 167008). Two-fold dilutions of L-Dopa, from 4.8 to 0.075 mM (final concentrations) were mixed (100 μl) with the enzyme solution. The enzymatic reaction was followed by measuring the absorbance at 475 nm every 30 s over 15 min, with shaking (150 rpm) between measurements. Temperature was kept constant at 30ºC. The values of K_M_ and v_max_ under these conditions were obtained from Lineweaver-Burk plots. For tyrosinase inhibition assays, each well of the microtiter plate was filled with 100 μl of a mushroom tyrosinase solution and the appropriate amount of the test substance dissolved in DMSO. The compounds were pre-incubated with the enzyme at 30ºC for 10 min. Subsequently, the enzymatic reaction was triggered by addition of 100 μl of L-Dopa (1.2 mM test concentration). Formation of dopachrome was followed by measuring the absorbance at 475 nm as described above. Kojic acid (Sigma-Aldrich) was used as positive control.

### Fluorescence spectroscopy analysis

Fluorescence measurements of tyrosinase solutions were performed using an Edinburgh Instruments LifeSpec II fluorescence spectrometer, exciting the complexes at 280 nm with an Edinburgh Instruments EPLED-280 subnanosecond pulsed diode source (typically pulse width at half maximum around 860 ps, repetition rate 10 MHz) and using Edinburg Instruments F900 acquisition software. A multichannel plate photomultiplier was used as the detector using single photon counting technique. Lifetime estimation was made using instrument response function (IRF) reconvolution analysis with FAST software by Edinburgh Instruments, providing a temporal resolution around 0.2 ns. A blank medium without enzyme was used to confirm that the collected fluorescence comes from the enzyme molecules and any influence of the fluorescence of the quencher and buffer components on the enzyme fluorescence is negligible. Enzyme solutions (2 mg ml^-1^) were prepared in phosphate buffer (50 mM, pH 6.5) in the presence of different concentrations of compound **16** (0–30 μM). A denatured enzyme sample was prepared by mixing the enzyme solution with an extremely high quencher concentration (1 mM), which provides a surfactant effect.

### Mussel foot retraction assay

The mussel *Mytilus galloprovincialis* was used as target organism. Individuals of 4.0–4.5 cm shell length were collected at intertidal rocky shore of the outer Ría de Vigo (NW Spain). Animals were kept in running seawater aquarium tanks with open-flow design for few days and fed daily by pulses with a mixture of the microalgae (Tahitian *Isochrysis* aff. *galbana*) and sediment from the seafloor below the mussel rafts.

Mussel foot retracting assay was conducted following the method reported by Hayashi and Miki [53]. Briefly, subsamples of individuals from the maintenance tanks were transferred to containers filled with 1-μm FSW. After 30 min, the posterior adductor muscle (PAM) was cut to open both animal´s valves as shown in S1A Fig, and byssus filaments were removed from their insertion with the soft tissues. Animals were disposed individually on flexible innocuous plasticine basis as a holder still for 15 min in FSW. The latter disposition avoided animal´s manipulation before and after each test solution was dripped directly on the foot of immobilized animals. FSW was removed and animals exposed their internal cavities in dry conditions for the dripping actions but maintaining a humidity layer over soft tissues.

Initially two different controls (negative and positive) were considered. As negative control, 2.5 μl of DMSO in 0.2-μm FSW was used. Only animals that did not react were considered further. After the negative control test, the animals were disposed back in 1-μm FSW for 15 min. With regard to the positive control, 1000 ppm of CuSO_4_ was used. For the latter case, only animals that did react to such positive control were used. After this second control, animals were disposed back in 1-μm FSW, this time for 25 min in order to remove completely any rest of copper. All animals considered for the experiment have accomplished the two controls satisfactorily.

Different concentrations (6.25, 12.5, 25, 50, 100 and 200 μM) of two test compounds, **3** and **16** were used. Stocks of 40 mM for the two compounds under investigation were prepared in DMSO. Posterior dilution actions to get the distinct concentrations desired were prepared with 0.2-μm FSW. Concentrations were tested in ascending order from 6.25 to 200 μM. Animals were kept in 0.2-μm FSW for 15 min at each time interval between sampling with the aim to wash the internal soft tissues. After each removal of seawater, animals were ready to use again.

Eight series of ten animals each were considered for retracting muscle foot analysis and each test compound. Each potential foot-reacting substance was individually dripped on the foot of the mussels (S1B Fig) and the animal’s reactions were noted. Activity was reported for each test compound concentration as the percentage of mussels showing reaction at several concentrations of the test compound according to the formula: (No. of reacted mussels / No. of total mussels) x 100. Final data are given as means ±SD.

## Results and Discussion

In this study, the antifouling profile of a collection of 20 alkyl triphenylphosphonium salts has been evaluated in detail. These compounds have a common triphenylphosphonium moiety and differ in the length of and chemical functionalities present in the alkyl chain (Fig 2). To that end, the compounds were assayed in a panel composed by target microscopic and macroscopic fouling species, as well as an acute toxicity model (*A*. *salina*), QS models (*C*. *violaceum*, *V*. *harveyi*), and an enzymatic model (tyrosinase). In the following sections, organized according to the kind of activity displayed, insights about their modes of action and structure-activity relationships are discussed.

### Biocidal activity

The biocidal profile of the compounds evaluated is summarized in Table 1. The following lines discuss the results showed therein. With respect to their bioactivity, a borderline can be established below C_7_ alkyl chains. These short-chain compounds were in general not active inhibiting bacterial or fungal growth with a few exceptions with *P*. *atlantica* and *C*. *lytica*. These products were also unable to inhibit the germination of *G*. *oxysperma* spores (only compounds **1** and **5** exhibited activity at 100 μM) and displayed little or null toxicity in *A*. *salina* nauplii, with the only exception of **5** that exhibited an unusually low LC_50_ of 17.2 μM. Apparently, the secondary amine bound to the phosphorus atom is the responsible for this activity, since it is the only difference between **5** and **7** (LC_50_ > 100 μM). Conversely, diatoms were more sensitive to the action of the products, and IC_50_ values in the range 8.2–81.5 μM were determined for this subset of compounds.

**Table 1:** Activity displayed by the tested compounds towards different fouling organisms.

Compound	*C*. *marina*	*P*. *atlantica*	*S*. *algae*	*V*. *alginolyticus*	*V*. *anguillarum*	*C*. *lytica*	*Alternaria* sp.	*Aspergillus* sp.	*Fusarium* sp.	*Amphora* sp.	*Cylindrotheca* sp.	*Navicula* cf. *salinicola*	*Nitzschia* sp.	*P*. *tricornutum*	*G*. *oxysperma*	*A*. *salina*
**1**	>100	>100	>100	>100	>100	>100	>100	>100	>100	19.5	9.8	10.6	18.7	16.8	100	>100
**2**	>100	>100	>100	>100	>100	>100	>100	>100	>100	43.4	23.7	50.2	40.2	>100	>100	>100
**3**	>100	9.5	>100	>100	>100	73.2	>100	>100	>100	>100	>100	62.2	>100	>100	>100	>100
**4**	>100	26.4	>100	>100	>100	>100	>100	>100	>100	>100	>100	>100	>100	>100	>100	92.7
**5**	>100	>100	>100	>100	>100	41.8	>100	>100	>100	42.5	12.6	8.2	11.9	81.5	100	17.2
**6**	>100	>100	>100	>100	>100	>100	>100	>100	>100	>100	21.3	>100	>100	>100	>100	>100
**7**	>100	>100	>100	>100	>100	>100	>100	>100	>100	>100	>100	>100	>100	>100	>100	>100
**8**	>100	28.6	>100	>100	>100	20.0	12.0	12.4	>100	37.6	10.6	6.4	20.8	92.2	100	20.4
**9**	21.0	6.6	8.6	69.7	87.0	2.6	7.4	10.0	31.4	6.2	1.9	2.7	3.6	11.2	50	2.0
**10**	8.0	5.2	10.4	35.5	18.3	2.6	5.2	11.7	21.0	3.2	1.0	2.3	2.5	1.9	50	1.4
**11**	6.1	3.0	5.4	32.9	17.0	1.1	3.5	5.1	5.3	3.3	1.0	1.5	2.3	1.7	50	1.8
**12**	5.1	1.1	3.0	26.3	27.5	1.7	1.2	7.5	6.6	4.8	1.0	1.3	2.2	1.2	50	2.4
**13**	5.0	1.2	3.6	11.3	5.7	1.7	1.6	4.8	6.5	4.5	1.7	1.8	2.2	1.0	50	0.9
**14**	5.3	2.3	1.2	12.7	1.6	2.0	0.7	5.3	5.1	2.1	4.5	1.2	3.5	1.5	50	1.8
**15**	5.5	2.9	3.8	5.6	2.7	1.9	2.9	5.2	4.2	3.1	2.2	2.8	3.6	1.1	50	2.8
**16**	9.9	6.8	5.4	16.9	5.3	1.5	1.4	5.2	9.3	2.6	4.7	1.3	4.9	1.3	50	1.8
**17**	>100	46.1	72.5	>100	31.8	11.3	1.3	10.2	8.8	4.2	8.0	1.2	7.6	7.4	>100	<0.5
**18**	>100	>100	>100	>100	>100	5.3	5.3	19.6	7.3	3.4	6.9	0.8	4.8	16.9	>100	<0.5
**19**	>100	>100	>100	>100	>100	>100	>100	>100	>100	75.5	24.1	>100	>100	>100	>100	>100
**20**	7.3	0.8	2.2	93.5	8.0	1.1	0.3	0.6	5.6	1.6	1.3	0.6	2.0	1.0	5	3.0
**TBTO**	34.2	46.6	6.4	38.6	27.5	0.5	0.6	3.0	6.7	5.7	0.4	6.3	2.8	1.7	5	9.2

Data represent the IC_50_ (μM) of three replicates. For *G*. *oxysperma*, the value indicated is the MIC.

A clear shift in terms of activity is observed for products with alkyl chains of C_7_ or above. In the antibacterial tests, the lowest IC_50_ values were generally achieved by compounds **12**–**14**. Indeed, the activity reached a maximum for alkyl chains between C_10_ and C_16_, with a rapid loss of activity for longer chains. The activity for this group of compounds was notably better than that displayed by TBTO. However, the presence of a carboxyl group at the end of the hydrocarbon chain (compound **19**) led to a total loss of activity, not only with bacteria but in all the bioassays conducted. Activity towards fungi followed an analogous pattern to that described for bacteria. In this case, the lowest IC_50_ values were recorded for compounds **13**–**15**. Interestingly, the presence of an additional triphenylphosphonium moiety (compound **20**) lowered the IC_50_ to values below that of TBTO. In diatoms, the decrease in the activity for very long alkyl chains was only evident in *Nitzschia* sp. Low-μM or even nM IC_50_ values (compounds **18** and **20**, *P*. *tricornutum*) were recorded. These results in microalgae contrast with the relatively high MICs (50 μM, compounds **9**–**16**) necessary to suppress macroalgal spore germination. Only **20** achieved an activity equivalent to that of TBTO. In *A*. *salina* tests, the activity increased stepwise with the length of the hydrocarbon chain. In fact, compounds **17** and **18** exhibited a very potent activity, with LC_50_ values below 500 nM, and all compounds with C_9_ alkyl chains or higher surpassed the activity of the TBTO control, with the abovementioned exception of compound **19**, which was inactive.

The mode of action of cationic biocides has been intensively described, primarily as antibacterial agents, and it is often depicted in six sequential stages: 1) adsorption on the bacterial cell wall, caused by electrostatic interaction between the negatively-charged bacterial surface and the cationic compound [9]; 2) diffusion through the cell wall; 3) attachment to the cell membrane; 4) lysis of the cell membrane; 5) leakage of the cytoplasmic contents and 6) cell death [54]. The ability to kill microbial cells by contact has promoted the use of quaternary ammonium and phosphonium compounds as disinfectants and antiseptics in solution over decades or, more recently, incorporated into materials surfaces and polymers for the avoidance of microbial biofilm formation [55].

**Fig 3:**
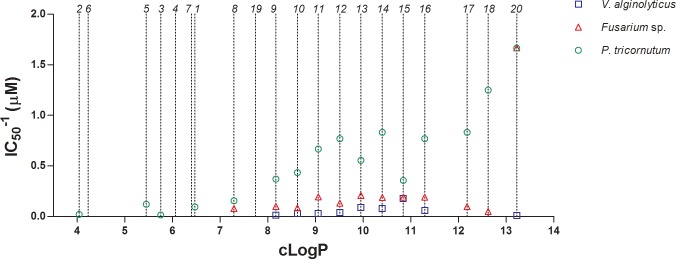
Correlation between the activity and the cLogP of the compounds evaluated in this study. Data correspond to three representative organisms: *Vibrio alginolyticus* (blue squares), *Fusarium* sp. (red triangles) and *Phaeodactylum tricornutum* (green circles). Dotted lines show the cLogP of each compound, whose ID. number is indicated above. Missing symbols indicate lack of activity.

There is a clear correlation between the length of the alkyl chain and the biocidal activity (Table 1 and Fig 3). This correlation was highly dependent on the target cell. This phenomenon has often been linked to the increased hydrophobicity of the compounds with longer alkyl chains and the subsequent decrease in the critical micellar concentration (cmc), thus facilitating a surfactant mode of action [19]. It should be recalled that the IC_50_ values determined for the products used in this study are well below their cmcs [56], thus cell death is unrelated to a surfactant activity. Fig 3 shows the activity of the compounds in function of their cLogP value in three tested microbial cells. For instance, for the Gram-negative bacterium *V*. *alginolyticus*, the optimal activity is achieved by compound **15**, whereas alkyl chains of C_18_ and above caused complete loss of activity. These findings are in fair agreement with previously reported data [57]. For the filamentous fungus *Aspergillus* sp., a similar trend is observed in the homologous series, although the compounds with the longest alkyl chains remained active. However, for the diatom *P*. *tricornutum* the activity improved as cLogP increased. This is likely due to the different nature and chemical composition of the microbial cell wall in each case. Nonetheless, cLogP alone is not the only factor affecting the biocidal effect of quaternary phosphonium compounds in solution. Two examples are evidenced in this study: **19** and **20**. The incorporation of a carboxyl moiety at the end of the alkyl chain (**19**) causes a decrease in cLogP from 9.06 (**11**) to 7.74 (**19**). This value is still above the ‘virtual threshold’ established by heptyl triphenylphosphonium bromide (**8**) as discussed previously. However, **19** is inactive, a fact likely derived from a different state of aggregation of the compound in solution. At alkaline pH (7–8), the carboxyl group exists as a carboxylate and may interact with the positively charged triphenylphosphonium moiety. As alkyl-triphenylphosphonium compounds need to trespass the biological membranes, this configuration leads to the inactivation of the compound. Conversely, **20** has two positive charges and interacts strongly with the negatively-charged cell membranes.

### Quorum sensing inhibition

Biofilm formation is a first stage in the biofouling process (Fig 1). The development of biofilms is a crucial step in the whole phenomenon as they alter the microtopograhy and physicochemical properties of the surface and, as a consequence of microbial metabolism and signaling, they change the chemical nature of the surface and produce an array of chemical cues that can deter, but more frequently attract, the arrival of secondary and tertiary colonizers [58].

QS, or bacterial intercellular communication, regulates bacterial gene expression as a function of population density [59]. The process relies on the production, excretion and detection of signaling molecules (generally, small molecules such as acyl homoserine lactones in Gram-negatives and small peptides in Gram-positives) so that, when a threshold extracellular concentration of AIs is achieved, bacterial gene expression is altered. Among the QS-regulated processes are biofilm formation and maturation [60–63]. There is clear evidence that the settlement of macrofouling organisms is modulated by the development of bacterial biofilms, as it is the case, for instance, of the polychaete *Hydroides elegans* [64,65], or *Ulva* species, whose zoospores can detect acyl homoserine lactones as positive cues for settlement [66]. For this reason, QS inhibitors have been proposed over the last few years as a mean to disrupt, or at least delay, biofouling [67,68]. QS inhibitors are an attractive antifouling treatment since they constitute a means of controlling biofilm formation without exerting a selective pressure (i.e. toxicity) on bacterial populations, thus avoiding bacterial resistance towards biocidal treatments. Indeed, bacterial cells in biofilms are up to 1000 times more resistant—or, more precisely, more tolerant- to chemical treatment than their planktonic counterparts [69].

**Fig 4:**
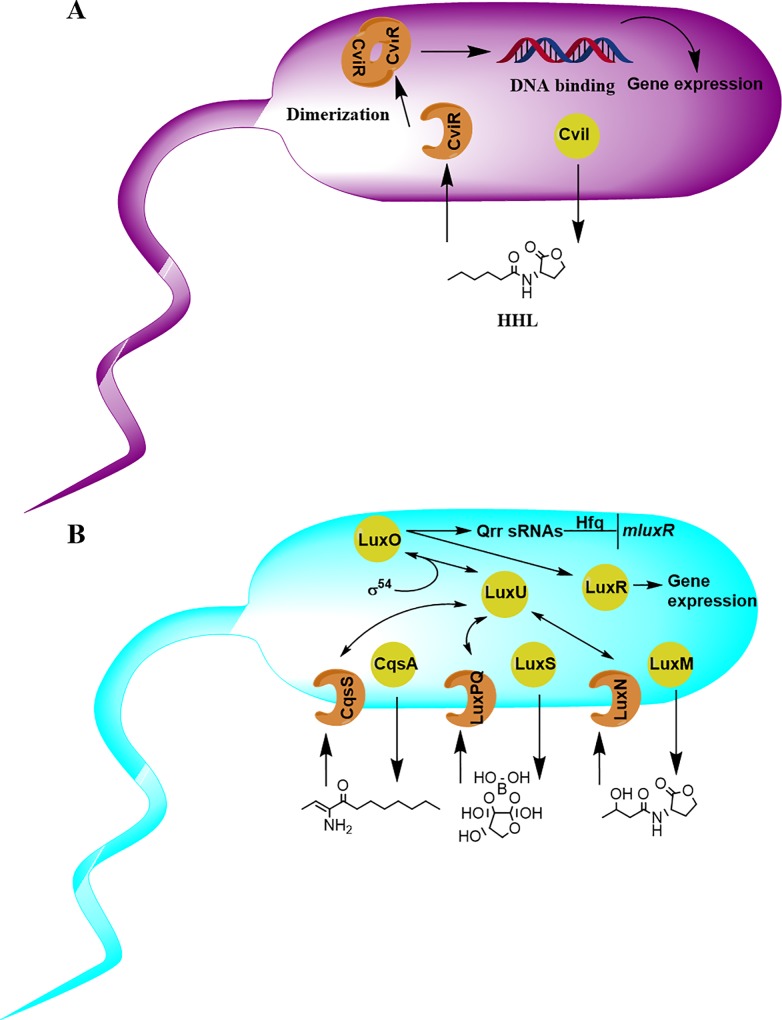
Quorum sensing circuits of *Chromobacterium violaceum* (A) and *Vibrio harveyi* (B). In *C*. *violaceum* ATCC 31532 (A), the synthase CviI produces the AI molecule HHL that is recognized by the cytoplasmatic receptor CviR. When bound to HHL, CviR dimerizes and binds DNA, leading to the expression of QS-regulated genes, including those involved in violacein production. In *V*. *harveyi* (B), three different AIs, synthesized by LuxM, LuxS and CqsA are recognized by the transmembrane two-component receptors LuxN, LuxPQ and CqsS, respectively. At low AI concentrations, these receptors act as kinases, phosphorylating LuxU and subsequently the σ^54^-dependent response regulator LuxO. The phosphorylated LuxO activate the transcription of Qrr sRNAs that together with the chaperone Hfq, destabilize the mluxR RNA. At high AI concentrations, the receptors switch to phosphatases and the expression of the master regulator LuxR is allowed.

To evaluate the effect of the alkyl triphenylphosphonium salts on QS-regulated phenotypes, an initial screening at the cut-off concentration of 100 μM was conducted with *C*. *violaceum* CVO26. The QS model of *C*. *violaceum* is relatively simple and consists of an acyl-homoserine lactone synthase (CviI) that produces the AI molecule *N*-hexanoyl homoserine lactone (HHL, strain ATCC 31532), or *N*-3-hydroxydecanoyl homoserine lactone (strain ATCC 12472) [70] which is recognized by the cytoplasmic receptor CviR (Fig 4). The reporter strain CVO26 is a CviI::mini-Tn5 mutant of strain ATCC 31532 and thus recognizes HHL. CviR is a DNA-binding transcription factor that activates the expression of the genes encoding the production of violacein, the characteristic purple alkaloid that gives name to the species [70].

**Fig 5:**
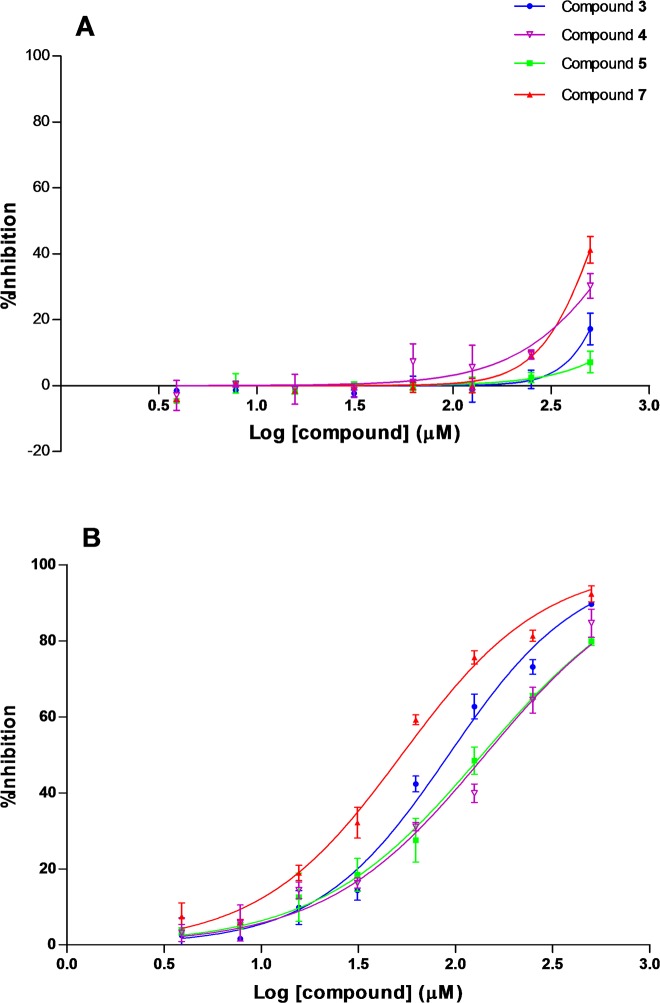
Dose-response curves for compounds 3–5 and 7 on *C. violaceum* CVO26 growth (A) and violacein synthesis (B). Data represent the mean ± SD (N = 3).

**Fig 6:**
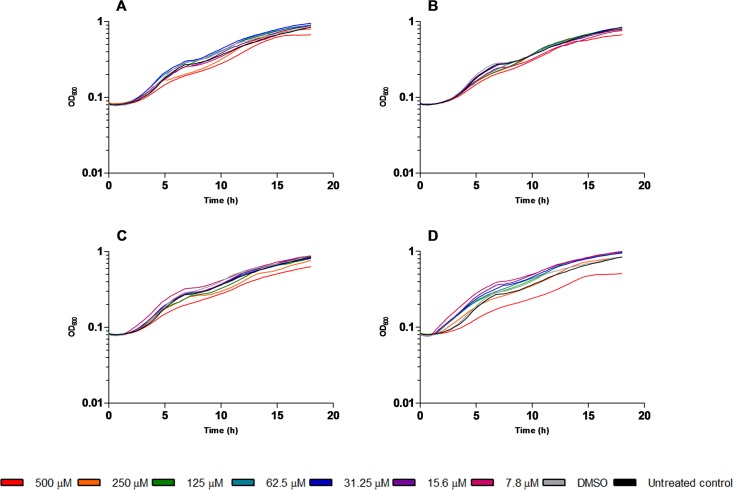
Growth curves of *C*. *violaceum* CVO26 in the presence of compounds 3 (A), 4 (B), 5 (C) and 7 (D). Serial two-fold dilutions of the compounds from 500 to 7.8 μM were tested. A detailed version of this Fig is provided (S2 Fig).

Interestingly, an inverse correlation between the length of the alkyl chain and the inhibition of the QS-regulated phenotype was found. Indeed, the inhibitions observed in violacein production for long alkyl chains (**10**–**18**, **20**) were clearly correlated to the biocidal effect exerted by these compounds, with ‘therapeutic ratios’ (IC_50_ for growth inhibition/IC_50_ for violacein inhibition) around 1, in general (Table 2). Note that, as the length of the hydrocarbon chain becomes shorter, this ratio increased (**8**–**9**). Compounds **1** and **2** were inactive. However, four compounds (**3**–**5**, **7**) displayed an attractive profile as QS inhibitors, with IC_50_ values for bacterial growth inhibition above the highest test concentration and consequently high therapeutic indexes (Table 2). To evaluate these compounds in further detail, 40-mM stock solutions were prepared in water to avoid the use of high concentrations of DMSO in the bioassays. As compound **7** has a poor solubility in water a 200-mM stock in DMSO was prepared. Serial two-fold dilutions (500–3.9 μM) were assayed. Consequently, the highest amount of DMSO used in the assay was 0.25% (v/v). The recorded IC_50_ values for growth inhibitions were all above 500 μM, whereas violacein inhibitions were in the range 52.9–142.2 μM (Table 2 and Fig 5). In terms of growth kinetics, concentrations up to 250 μM of these compounds did not display any significant effect (Fig 6 and S2 Fig). Growth inhibitory effects are only observed at 500 μM, particularly for compound **7** (Fig 6D).

**Table 2:** Half-maximal inhibitory concentrations (μM) for the tested compounds on the growth and violacein production of *C*. *violaceum* CVO26.

Compound	IC_50_ (Growth inhibition)	IC_50_ (Violacein inhibition)	Ratio (GI/VI)
**1**	>100	>100	-
**2**	>100	>100	-
**3**	>500	92.3	>5.4
**4**	>500	142.2	>3.5
**5**	>500	136.8	>3.7
**6**	>100	>100	-
**7**	>500	52.9	>9.5
**8**	52.5	19.0	2.8
**9**	16.0	7.6	2.1
**10**	11.9	7.7	1.6
**11**	4.9	3.5	1.4
**12**	10.3	6.6	1.6
**13**	3.2	3.9	0.8
**14**	2.3	4.6	0.5
**15**	2.6	1.2	2.2
**16**	3.2	4.2	0.8
**17**	7.0	7.0	1.0
**18**	40.1	35.2	1.1
**19**	>100	>100	-
**20**	12.8	16.7	0.8

The index on the right column is calculated as the ratio between the IC_50_ value for growth inhibition (GI) and that for violacein (VI).

To check whether the compounds were able to thwart other QS-regulated phenotypes and gain knowledge on their molecular targets, products **3**–**5** and **7** were evaluated in the more complex QS model of *Vibrio harveyi* (Fig 4B). In *V*. *harveyi*, three AIs are synthesized by LuxM (produces the HAI-1 signal, an acyl homoserine lactone), LuxS (AI-2 signal, a furanosyl borate diester) and CqsA (CAI-1 signal, an α-amino ketone), respectively. These signals are believed to mediate intra-species, inter-species and intra-genera communication, respectively [71]. These AIs are detected by three membrane sensors: LuxN, LuxPQ and CqsS, respectively. At low AI concentrations, the receptors phosphorylate the σ^54^-dependent response regulator LuxO by means of the phosphotransferase LuxU, which activates the production of Quorum-regulatory RNAs (Qrr sRNAs), four of which, together with the chaperone Hfq, target and destabilize the mRNA that encodes the master regulator LuxR. On the contrary, at high AI concentrations, the QS receptors dephosphorylate LuxO via LuxU, allowing the expression of more than 100 genes, those encoding luciferase amongst them [72].

**Fig 7:**
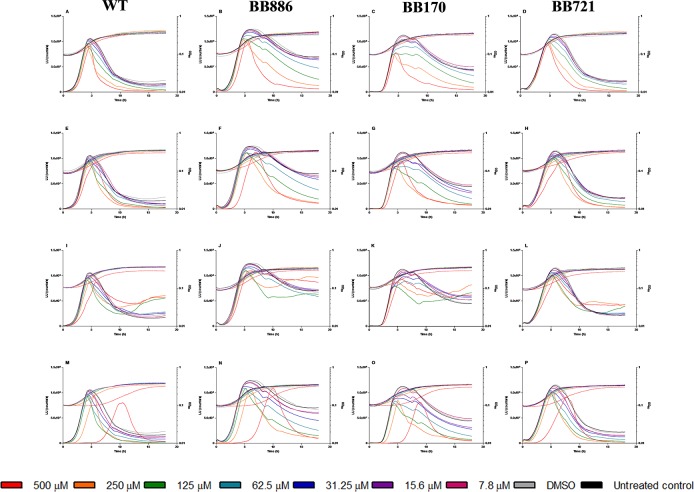
Bioluminescence (solid lines) and growth curves (dotted lines) for compounds 3–5 and 7 in *V*. *harveyi*. Compound **3** (A-D); compound **4** (E-H); compound **5** (I-L); compound **7** (M-P). Serial two-fold dilutions of the compounds from 500 to 7.8 μM were tested.

Compounds **3**–**5** and **7** were evaluated in *V*. *harveyi* WT and three mutant strains: BB886 (luxPQ::Tn5Kan), unable to detect AI-2; BB170 (luxN::Tn5Kan), unable to detect HAI-1; and BB721 (luxO::Tn5lacZ), a LuxO null mutant that produces maximal luminescence per cell constitutively. In order to take into account the cumulative effects of the compounds, bioluminescence and growth were measured simultaneously every 15 min over 18 h (Fig 7). Accordingly, IC_50_ values for bacterial luminescence and growth were obtained by integration of the areas under the curves for each tested dose (Table 3). For clarity of the graphs, error bars has been omitted in Fig 7 and only the mean curves are shown. A detailed version of this Fig. showing the dispersion of the results is provided as S3 Fig. Several deductions can be inferred from these data:

**Table 3:** IC_50_ values (μM) for luminescence and growth inhibitions caused by compounds 3–5 and 7 in *V*. *harveyi* WT and reporter strains.

Compound	Luminescence				Growth
	WT	BB886	BB170	BB721	
**3**	296.7	265.2	251.0	348.5	>500
**4**	>500	306.9	225.8	>500	>500
**5** *	>500	>500	>500	>500	>500
**7**	205.1	179.4	128.6	276.4	>500

*Non-monotonic response.

**Fig 8:**
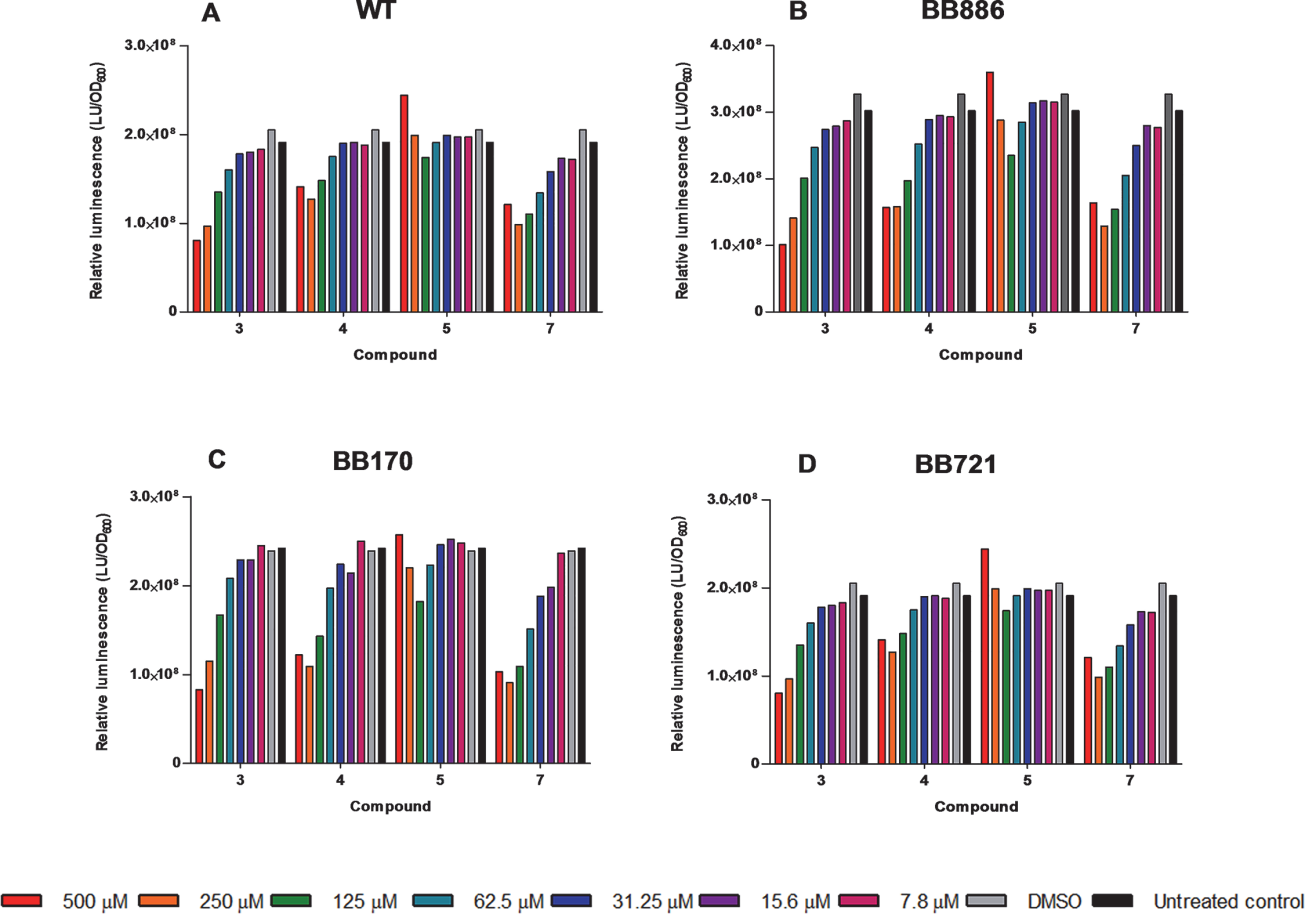
Relative luminescence ratios (Light units/OD_600_) for compounds 3–5 and 7 in *V*. *harveyi*. WT (A), BB886 (B), BB170 (C) and BB721 (D). Serial two-fold dilutions of the compounds from 500 to 7.8 μM were tested.

First, regarding the growth curves, some toxicity was observed at the highest dose (500 μM), evidenced as a growth delay, for compounds **4** and **5** (Fig 7E–7H and 7I–7L). This effect was more prominent for compound **7** (Fig 7M–7P) but notably lower for compound **3**, which indeed did not cause any substantial effect on bacterial growth (Fig 7A–7D). In addition, inhibitions in bioluminescence were not strictly proportional to the inhibitions impaired on bacterial growth, evidenced by the bioluminescence:growth ratios shown in Fig 8. Thus, toxicity does not explain by itself the effects observed in the luminescent phenotype. Second, according to the IC_50_ data for the different signaling pathways presented in Table 3, although the IC_50_ values obtained for inhibitions in the AI-2-mediated QS were slightly lower than those obtained for that mediated by HAI-1, there seem to be no clear preference of the compounds for one or another receptor, further confirmed by the luminescence inhibitions observed for the LuxO null mutant. Since *V*. *harveyi* BB721 is constitutively bright, we deduce that any luminescence inhibition in this strain, if observed, should be due to interaction of the test substance with elements downstream LuxO (although strictly, an inhibition caused by direct interaction of the compounds with the luciferase enzyme cannot be discarded with these data; the possibility of multiple targets cannot be discarded either). Third, it is particularly evident a non-monotonic dose-response relationship for compound **5** (Fig 7I–7L and Fig 8) which is in marked contrast with the monotonic trend of the other three phosphonium bromides. This non-monotonic behavior is more evident in the HAI^-^ and AI2^-^ mutants (Fig 7J and 7K), and less accused in the WT and LuxO^-^ strains (Fig 7I and 7L), suggesting a preferential interaction with the LuxN and LuxPQ receptors. Since these are membrane-bound proteins, this evidence agrees with the mode of action of triphenylphosphonium compounds, which target and trespass the cell membranes.

There are very few reports of QS inhibition by quaternary ‘-onium’ salts. For instance, quaternary ammonium compounds such as calmidazolium has been proven to interfere with QS in *V*. *harveyi* [73], whereas Peach and co-workers highlighted two miltefosine-related compounds as hits in a high-throughput screening of biofilm inhibitors in *V*. *choleare* [74]. In that case, however, the authors did not study if biofilm inhibition was caused by QS disruption or through any other mechanism. To the best of our knowledge, this is the first report of QS disruption by phosphonium compounds. In the present study, compounds **3**–**5** and **7** did not cause a substantially different response in either the LuxN receptor nor in the LuxPQ receptor. Evidence of QS disruption downstream LuxO further supports the existence of more than one target in the QS signaling circuit. Interestingly, the effect of compound **5** followed a non-monotonic trend. This kind of response was unexpected for a QS disruptor, but it is not uncommon in pharmacology, in particular for endocrine disruptors and hormones [75]. Among the causes attributed for this kind of response, one of the most common is nonselectivity [75]. Even though evidence of QS disruption, unrelated to toxic effects, is deduced from the present findings, the precise mechanisms describing how these compounds thwart bacterial cell-to-cell communication remain to be elucidated. It is worth to recall that, since quaternary phosphonium compounds have shown a promising profile as therapeutics and drug carriers [76–82], the present findings immediately suggest a potential use as coadjuvants in antibiotherapy or other antibacterial treatments (e.g. luminescent vibriosis in aquaculture [83]).

### Tyrosinase inhibition

Tyrosinases (polyphenol oxidases) are ubiquitous enzymes whose function is the catalysis of the hydroxilation of monophenols to *o*-monophenols, and the subsequent oxidation of the latter to *o*-quinones [84,85]. Tyrosinases play a key role in many biochemical processes, from the biosynthesis of pigments such as melanin to sclerotisation of insect cuticules or the production of biological adhesives such as those of barnacles and mussels [85,86]. Mussels become attached to rocks in the intertidal zone through the production of adhesive threads termed byssi. Byssogenesis occurs in three stages [87]: 1) the mussel explores a surface with a muscular organ termed ‘foot’ and, when a suitable place is found, the mussel cleans the surface of unattached matter and gets prepared for byssal secretion with contractional movements of the foot; 2) the foot is pressed tightly to the surface and a pull of adhesive proteins rich in DOPA residues is secreted, forming a byssal thread and a terminal plaque within few minutes; 3) the foot is retracted and the process is repeated to form more threads (S1 Video). Tyrosinases are present in the proteinaceous secretions of the foot glands and are involved in the curation (cross-linking) of the polymeric adhesive through the oxidation of DOPA (post-transcriptionally conversed to tyrosine) to *o*-quinone [87,88].

Tyrosinase inhibition is thus a key target for the laboratory testing of antifouling molecules. Although *Mytilus* sp. phenoloxidase differs to some extent from that of the widespread commercial source (*Agaricus bisporus*), the latter was used for the *in*-*vitro* testing of the phosphonium compounds since a) it is non-expensive and commercially available, thus making it suitable for screening purposes, and b) it has been previously proposed for antifouling testing [89]. Under our experimental conditions, the kinetic parameters K_M_ and v_max_ obtained for the catalytic conversion of L-Dopa to dopaquinone were 0.8–1.0 mM and 0.07–0.09 AU min^-1^. As Table 4 shows, only compounds with alkyl chains of C_12_-C_19_ exhibited IC_50_ values below the cut-off concentration of 100 μM. A steep shift in the activity is observed for lengths above C_14_, with a performance similar to that of the known tyrosinase inhibitor kojic acid. However, compound **20** did not display relevant inhibitions at the tested concentrations. Thus, it is deduced that the inhibitory activity is caused by the interaction of the alkyl chain with the enzyme, which is reverted by the presence of a second cationic moiety.

**Table 4:** Tyrosinase IC_50_ values (μM) for the compounds tested in this study.

Compound	IC_50_ (μM)
**1–11**	>100
**12**	94.5
**13**	78.9
**14**	34.7
**15**	28.4
**16**	17.5
**17**	15.0
**18**	12.3
**19**	>100
**20**	>100
**Kojic acid**	16.5

**Fig 9:**
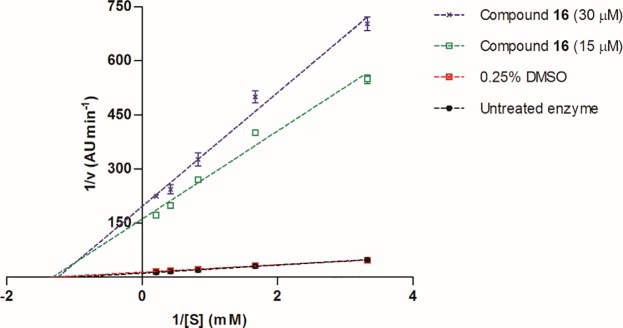
Lineweaver-Burk plots for tyrosinase inhibition in the presence of compound 16. Data represent the mean ± SD (N = 3).

Compound **16** was selected to further characterize the type of inhibition displayed by the triphenylphosphonium salts. Lineweaver-Burk plots of the inhibited enzyme by a dose around the IC_50_ (15 μM) and double (30 μM) in comparison to the uninhibited enzyme yielded a group of straight lines that converged at their intersection with the *x* axis (Fig 9). This pattern corresponds to a non-competitive inhibition, in which the inhibitor has the same affinity for the free enzyme than for the enzyme-substrate complex. These results suggest that compound **16** inhibits tyrosinase activity by causing a conformational change derived from its binding to an allosteric site.

**Fig 10:**
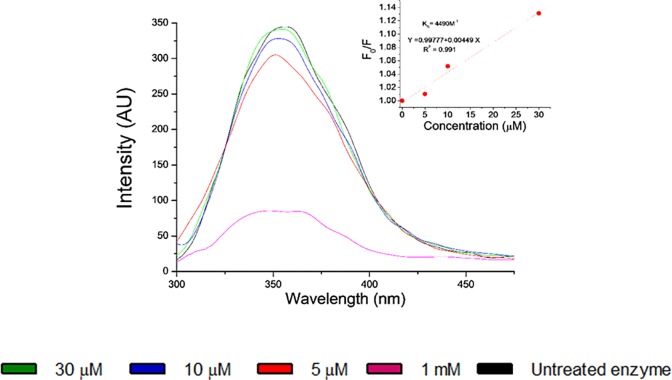
Fluorescence spectra of tyrosinase under 280 nm excitation at different concentrations of 16. The denatured and uninhibited enzyme were included as controls. Inset: Stern-Volmer plot of the fluorescence quenching.

Fluorescence of enzymes is very sensitive to conformational changes of the protein structure. The interaction of tyrosinase inhibitors can be evaluated by the analysis of the intrinsic tyrosinase fluorescence when tyrosinase and inhibitor (quencher) molecules are mixed in a solution. In particular, we have evaluated the effect of the tyrosinase inhibitor **16**. Quenching of tyrosinase fluorescence is expected as a consequence of conformational alterations induced by the quencher [90,91]. Tryptophan residues are basically responsible of the tyrosinase fluorescence when the compound is optically excited at around 280 nm. Fig 10 shows the fluorescence emission spectra of tyrosinase enzymes mixed with different quencher concentrations. The quencher concentrations chosen in this study ranged from zero (native enzyme) to 30 μM. This upper limit is well below the cmc threshold, which is estimated to be around 100 μM [56]. In this way, we assure that the reported tyrosinase fluorescence quenching is due to enzyme-inhibitor interaction and it is not related to any detergent-like side effect. In addition to this, a denatured enzyme sample was included in the study for comparison purposes. Quenching of the fluorescence intensity is observed as the concentration of the inhibitor increases. In some cases, a red-shift is expected in the maximum of the emission wavelength, which is associated to a higher exposure of the tryptophan residues in the distorted protein structure to the polar environment of the solvent [92]. However, there is no significant red-shift in the emission spectra, which indicates that the tryptophan residues are not very much exposed to the solvent environment.

Fluorescence quenching can be described by the Stern-Volmer equation:

$$\frac{F_{0}}{F}=1+K_{SV}\left[Q\right]$$ (1)

where F_0_ and F are the fluorescence intensities before and after the addition of the quencher, respectively, [Q] is the concentration of the quencher, and K_SV_ is the Stern-Volmer quenching constant, which indicates the sensitivity of the enzyme to the quencher. A linear dependence of the ratio F_0_/F on the quencher concentration can be observed in the inset of Fig 10. This is normally an indication of a single class of fluorophore, tryptophan residues, which are equally accessible to the quencher. The K_SV_ constant of about 4490 M^-1^ has been obtained from the best fitting of the Stern-Volmer plot to a linear equation. Two possible quenching mechanisms provide linear Stern-Volmer plots. On the one hand, collisional quenching of the fluorescence occurs when the quencher diffuses to the fluorophore during the lifetime of its excited state. The excited molecule returns to its ground state without emission of radiation due to contact with the quencher. This is a dynamic process, in which there is no permanent distortion of the protein. On the other hand, static quenching happens when a molecular complex is formed between the fluorophore and the quencher. In order to distinguish which of the two mechanisms is responsible of the tyrosinase fluorescence quenching, lifetime measurements can be performed.

In the case of collisional quenching (dynamic quenching) the lifetime of the excited state of the fluorophore decreases with the quencher concentration according to the following equation:

$$\frac{\tau_{0}}{\tau }=1+K_{SV}\left[Q\right]$$ (2)

Consequently,

$$\frac{F_{0}}{F}=\frac{\tau_{0}}{\tau }$$ (3)

This means that a shortening of the lifetime equal to that of the fluorescence intensity shoud be expected.

However, if static quenching is occurring, the enzyme-quencher complexes are non-fluorescent. This means that the fluorescence detected comes from proteins which have not interacted with the quencher and, consequently, their lifetime remains the same.

**Fig 11:**
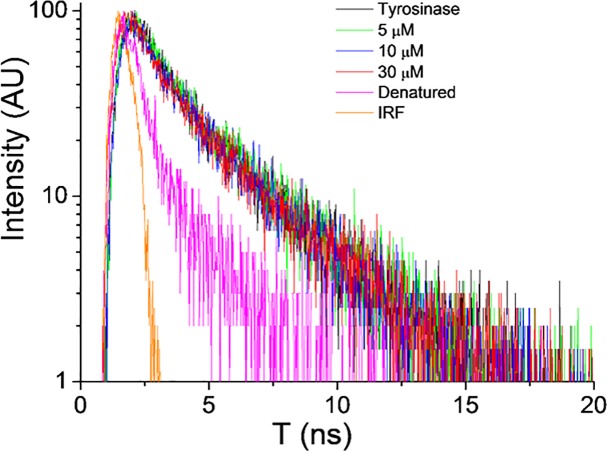
Decay of the fluorescence of tyrosinase tryptophan residues at different concentrations of compound 16. IRF is the instrumental response function.

The decay of the PL has been measured under excitation at 280 nm and detection at the maximum emission wavelength (Fig 11). A similar exponential decay curve has been measured for the native enzyme and for the enzyme-inhibitor solutions up to the highest quencher concentration used (30 μM). This result indicates that static quenching is responsible of the fluorescence quenching observed. When the data are fitted to an exponential decay curve the best fit is obtained for a lifetime value of about 2.3 ns. In contrast, a significant drop of the fluorescence lifetime to 0.75 ns is measured for the denatured enzyme.

The fact that the lifetime of the enzyme-quencher solutions is the same than that of the native enzyme indicates that the quenching of the fluorescence is due to a static process, which occurs by the formation of a permanent enzyme-quencher complex which is non-fluorescent. The short decay of the denatured protein is probably due to the unfolding of the enzyme, which exposes the tryptophan residues to the solvent environment introducing new non-radiative relaxation mechanisms, which are responsible of the shortening of the lifetime and decrease of the intensity of the fluorescence.

### Mussel foot retraction assay

At this point, two groups of triphenylphosphonium compounds can be distinguished: those inhibiting QS-regulated phenotypes, with a non-toxic mode of action (**3**–**5**, **7**) and those with a broad-spectrum biocidal and tyrosinase inhibitory activity (**12**–**18**). To investigate the behavior of both kinds of compounds as mussel repellents, a representative compound of each class was selected to be tested in *M*. *galloprovincialis*. Thus, compounds **3** and **16** were selected for further evaluation. As described before, mussels explore substrata with their feet before byssal formation. Consequently, substances causing a repellent response (evidenced by foot retraction) are likely to deter mussels from settling.

**Fig 12:**
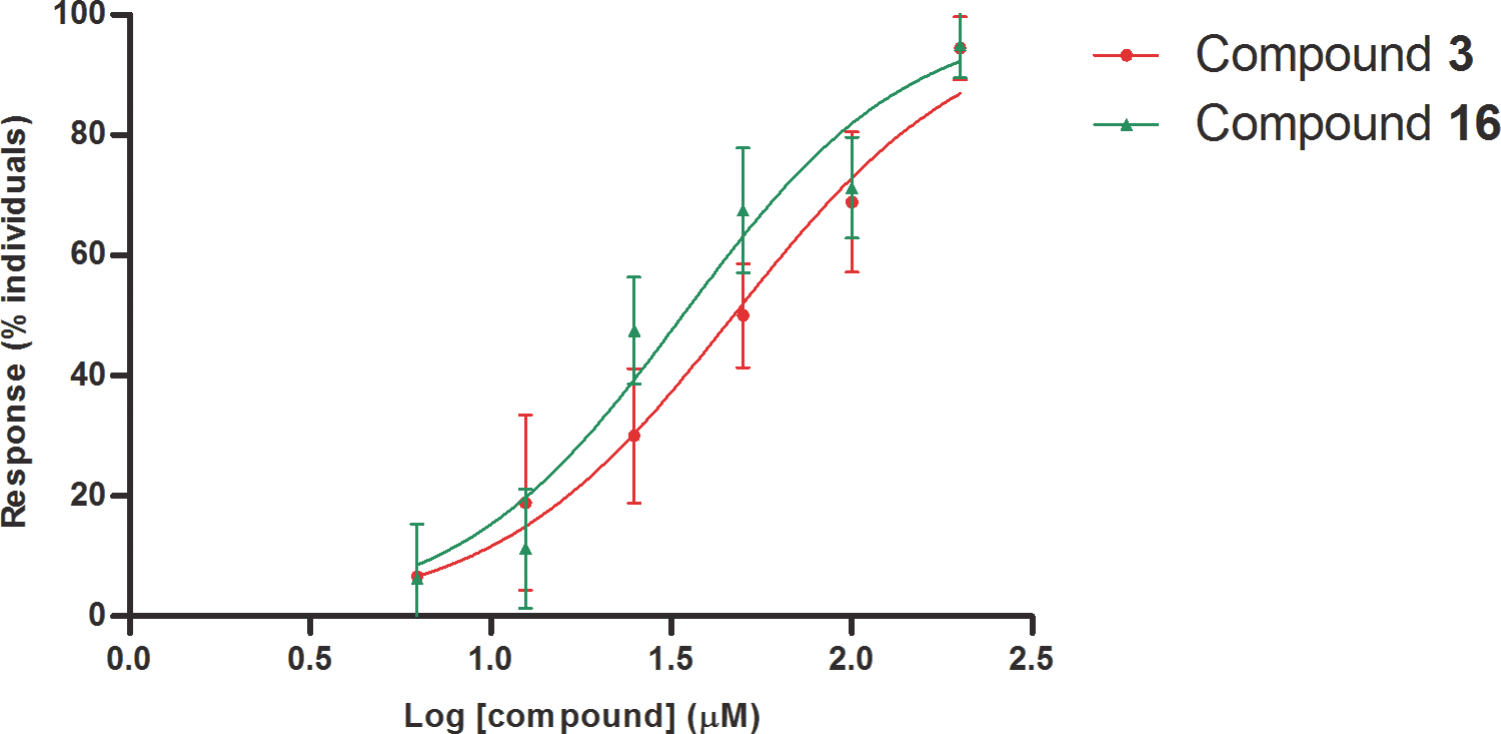
Dose-response curves for compunds 3 and 16 in mussel (*Mytilus galloprovincialis*) foot retraction assays.

Both **3** and **16** caused rather similar mussel foot retraction behaviors, with a clear dose-dependent effect (Fig 12). The highest test concentration (200 μM) caused the strongest effect for both tested compounds (94–95%). Mean increments between consecutive increasing tests on the whole range of concentrations represented approximately two-fold increase for both compounds with maxima of four-fold and three-fold increase from 12.5 to 25 μM and 6.25 to 12μM for both **16** and **3**, respectively. The concentration of both compounds required to cause foot repulsion in 50% of mussels was 47 and 34 μM for **3** and **16**, respectively (Fig 12). Visual evidence of these results in comparison to the positive (CuSO_4_) and negative (FSW) control can be observed in S2–S4 Videos.

## Conclusions

**Fig 13:**
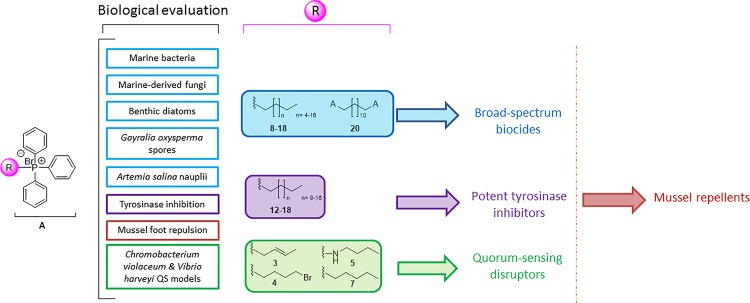
Summary of this study. The different colors highlight the main bioactivities and the structure-activity relationships of the tested triphenylphosphonium salts.

In this study, the antifouling profile 20 triphenylphosphonium salts has been comprehensively evaluated (Fig 13). The activity displayed by these compounds fall into four main categories:

1. Broad-spectrum biocides against marine microorganisms, macroalgal spores and invertebrates. These compounds interacted strongly with the biological membranes due to their high lipophilicity (> C_7_) or positive charge (**20**).
2. Tyrosinase inhibition, caused by compounds with alkyl chains above C_11_. This activity was notably higher for compounds **16**–**18**, with IC_50_ values similar to that of kojic acid, in the low-micromolar range. The type of inhibition was non-competitive. Fluorescence spectroscopy analyses confirmed that the inhibition was due to a static process, caused by the formation of a permanent enzyme-quencher complex.
3. QS inhibition. Four compounds in this series (**3**–**5** and **7**) exhibited a promising behavior as QS disruptors in two bacterial models: *C*. *violaceum* and *V*. *harveyi*. These compounds were non-toxic to bacteria at inhibitory concentrations and, in particular, compound **3** did not exert any toxic effect up to 500 μM. The evaluation in *V*. *harveyi* mutants suggests non-specificity, likely with multiple molecular targets, including elements downstream LuxO.
4. Mussel foot repellents. Compounds **3** (a QS inhibitor) and **16** (a broad-spectrum biocide and potent tyrosinase inhibitor) were tested in mussel foot retraction assays with *Mytilus galloprovincialis* as test organism. Both compounds exhibited a similar behavior, with effective doses of 47 and 34 μM, respectively.

Recall that compounds with negatively-charged functionalities at the end of the alkyl chain (**6**, **19**) were inactive, probably as a consequence of a different arrangement in solution.

The findings reported in this study widen the scope of use of triphenylphosphonium compounds as antifouling additives. However, it is worth to recall that the activities reported herein have been demonstrated with molecules in solution and it remains to test whether they are able to exhibit the same properties in functionalized material surfaces for antifouling protection. Since their biocidal properties has been exploited over decades to confer antimicrobial properties on different kinds of materials, it is likely that the new activities presented herein for this family of compounds will be retained as well.

Whereas the use of biocides is environmentally safe only when they are immobilized (e.g. functionalized surfaces) or they are easily biodegraded and released in a controlled way (e.g. self-polishing coatings), the use of non-toxic inhibitors of key processes for the biological colonization of material surfaces is more versatile and desirable. In this regard, it is particularly prospective the discovery of the ability of certain triphenylphosphonium compounds to disrupt bacterial cell-to-cell communication without exerting significant toxicities. We are aware that these properties could be also exploited in other fields not necessarily related to marine antifouling protection.

The promising profile of these non-toxic compounds was reinforced by the similar response that compound **3** caused in mussel foot retraction tests in comparison to compound **16**, which would implicate such non-toxic product selection for fouling impact analysis as a powerful strategy in terms of environmental impact versus broad-spectrum product efficiency balance. Those innocuous products for the biological environment that exert powerful repelling actions on the mussel feet (at least comparable to other components with high biocidal action) might be desirable for dealing with fouling impact. Nonetheless, it is necessary to keep in mind the gap between the antifouling impact and the mussel foot-repelling actions we are checking, which are more comparable to a negative chemotactic response rather than an inhibitory effect on mussel attachment.

Overall, this study re-focus on the antifouling properties of alkyl triphenylphosphonium compounds from a different perspective, including new biological targets such as tyrosinase inhibition or bacterial intercellular communication. As far as we are aware, this study reports for the first time the QS inhibitory properties of phosphonium compounds, which deserve a particular in-depth evaluation.

## Supporting Information

S1 Fig Arrangement of *M*. *galloprovincialis* individuals employed in foot retraction assays. The posterior adductor muscle is cutted to open both valves (A), and the foot-retracting assay is conducted by dipping the test solutions onto the animal’s feet (B).
(TIF)

S2 Fig Growth curves of *C*. *violaceum* CVO26 in the presence of compounds 3 (A), 4 (B), 5 (C) and 7 (D). Serial two-fold dilutions of the compounds from 500 to 7.8 μM were tested. Data represent the mean ± SD (N = 3).
(TIF)

S3 Fig Bioluminescence (solid lines) and growth curves (dotted lines) for compounds 3–5 and 7 in *V*. *harveyi*. Compound **3** (A-D); compound **4** (E-H); compound **5** (I-L); compound **7** (M-P). Serial two-fold dilutions of the compounds from 500 to 7.8 μM were tested. Data represent the mean ± SD (N = 3).
(TIF)

S1 File General methodological information and spectroscopic data of the compounds synthesized for this study. (DOCX)

S1 Video Time-lapse sequence of byssal thread formation. The sequence, that covers 4–5 h of continuous recording, shows clearly how the mussel extends its foot to explore the surface and produces a byssal thread.
(MP4)

S2 Video Mussel foot-retraction response to FSW (negative control). (MP4)

S3 Video Mussel foot-retraction response to 1000 ppm CuSO_4_ (positive control). (MP4)

S4 Video Mussel foot-retraction response to 200 μM of compound 3. (MP4)
