# Supplementary figures and images for: From Broad-Spectrum Biocides to Quorum Sensing Disruptors and Mussel Repellents: Antifouling Profile of Alkyl Triphenylphosphonium Salts
Source: PLoS One. 2015 Apr 21;10(4):e0123652. doi: 10.1371/journal.pone.0123652 (PMC4405350; doi:10.1371/journal.pone.0123652)

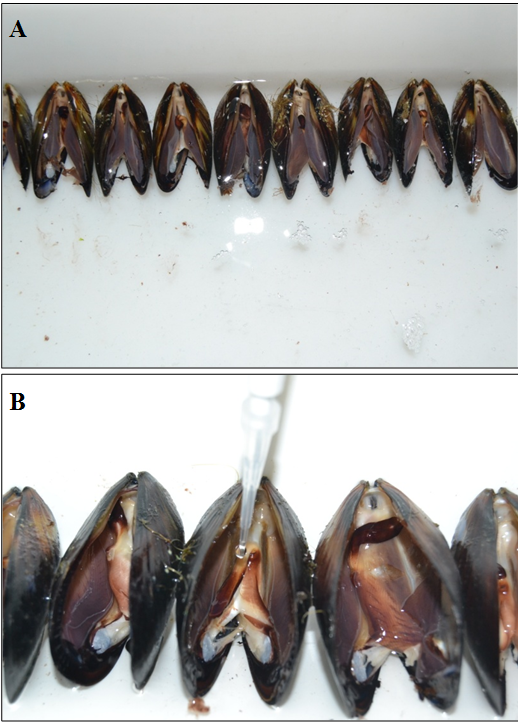

Supplement: S1 Fig — The posterior adductor muscle is cutted to open both valves (A), and the foot-retracting assay is conducted by dipping the test solutions onto the animal’s feet (B). (TIF) [file pone.0123652.s001.tif]

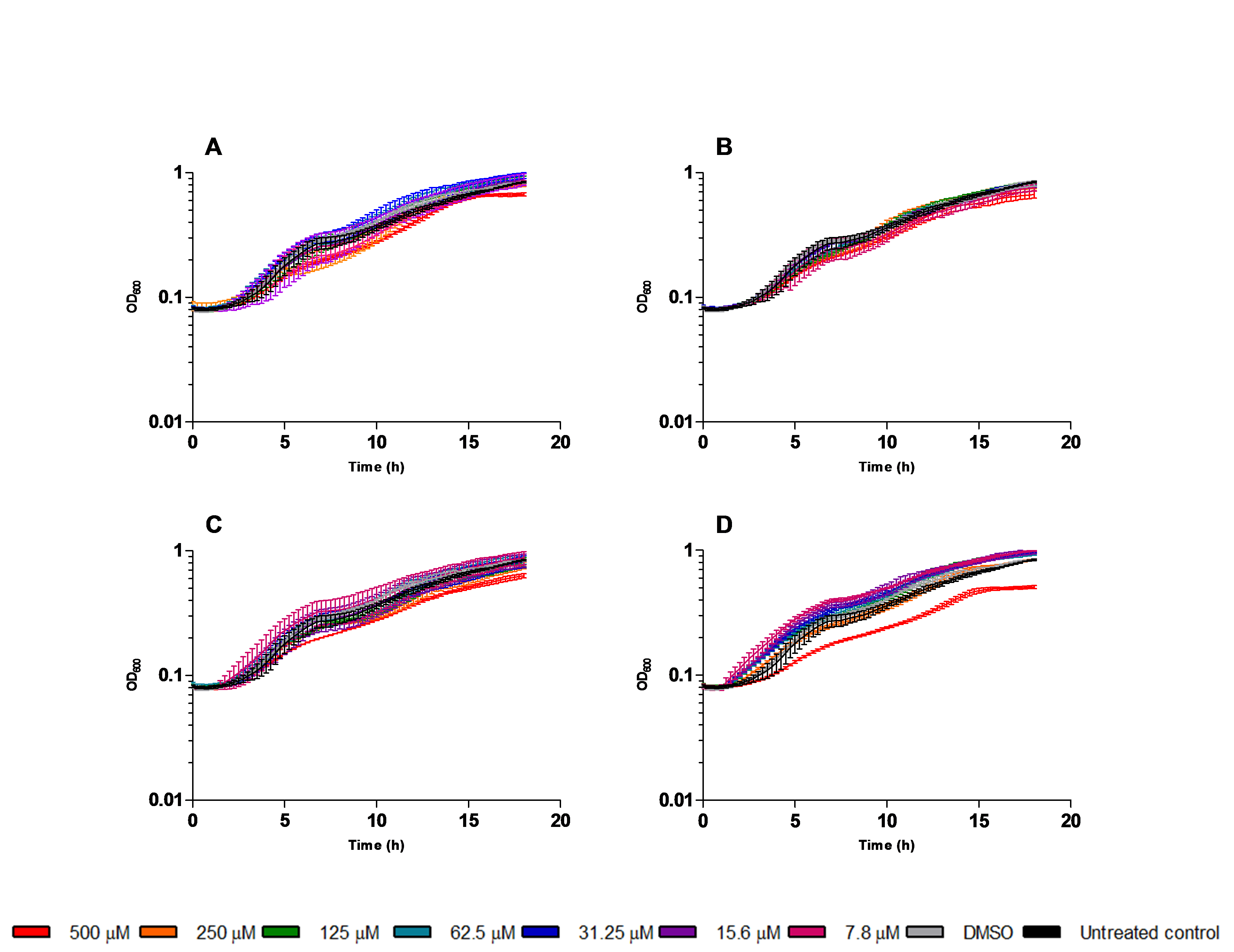

Supplement: S2 Fig — Serial two-fold dilutions of the compounds from 500 to 7.8 μM were tested. Data represent the mean ± SD (N = 3). (TIF) [file pone.0123652.s002.tif]

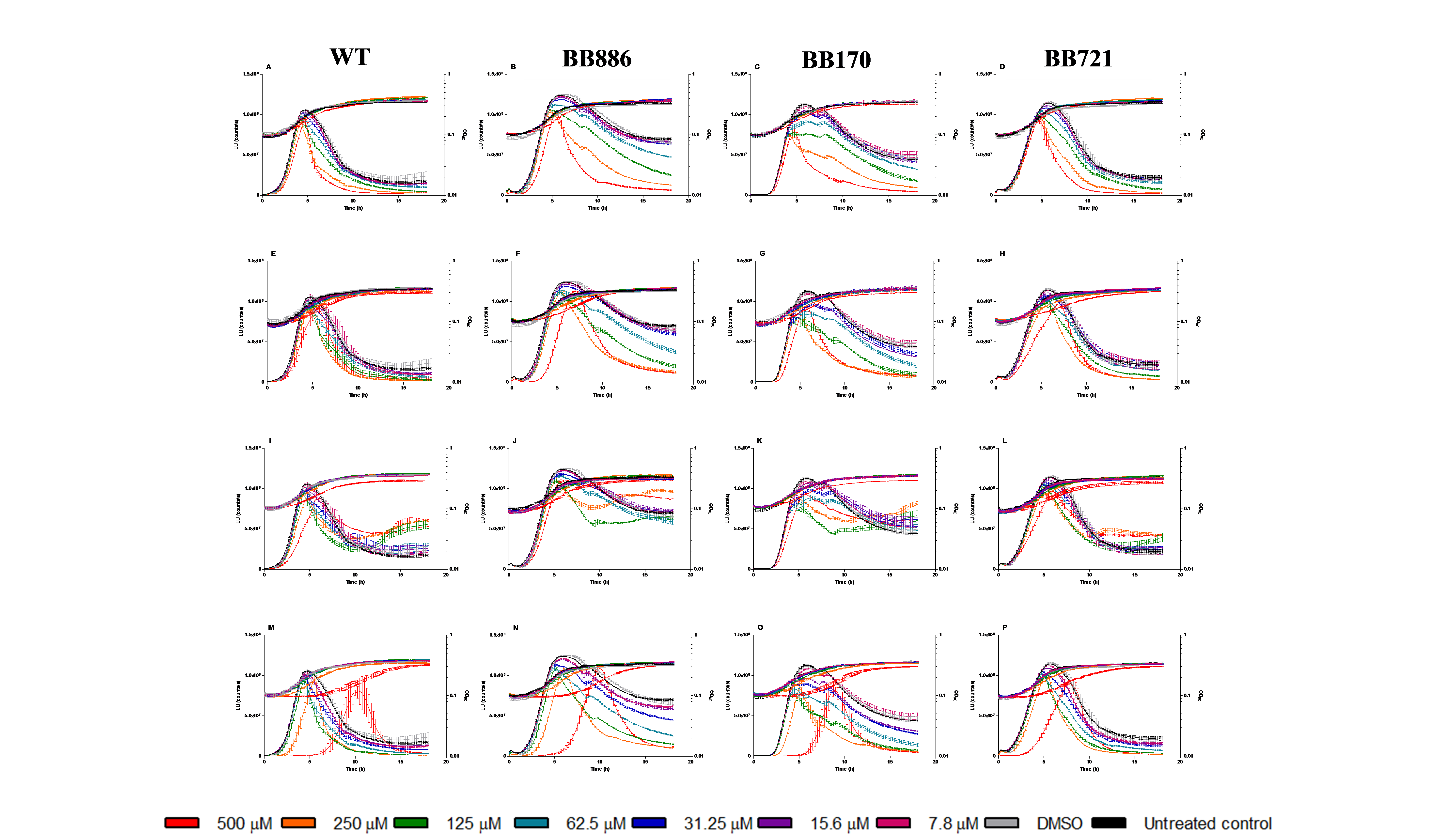

Supplement: S3 Fig — Compound 3 (A-D); compound 4 (E-H); compound 5 (I-L); compound 7 (M-P). Serial two-fold dilutions of the compounds from 500 to 7.8 μM were tested. Data represent the mean ± SD (N = 3). (TIF) [file pone.0123652.s003.tif]
