# Supplementary material for: From Broad-Spectrum Biocides to Quorum Sensing Disruptors and Mussel Repellents: Antifouling Profile of Alkyl Triphenylphosphonium Salts
Source: PLoS One. 2015 Apr 21;10(4):e0123652. doi: 10.1371/journal.pone.0123652 (PMC4405350; doi:10.1371/journal.pone.0123652)
Supplement: S1 File — (DOCX) [file pone.0123652.s004.docx]

**General Information**

NMR spectra were performed on Bruker AVANCE 500 MHz instruments at 300 K, and coupling constants are given in Hz. COSY, HSQC and HMBC experiments were performed using standard pulse sequences.  Data were processed using Topspin or MestRe software. All reagents were commercially available and used as received. All solvents were dried and distilled under Ar immediately prior to use, or stored appropriately. Reactions were monitored by TLC. Flash chromatography was performed with silica gel (230–400 mesh) as the stationary phase and mixtures of *n*-hexane and AcOEt, in different proportions given in each case, as the mobile phase. Mass spectra were recorded on a VG AutoSpec FISON spectrometer. TLC was performed on AL Si gel Merck 60 F254, and TLC plates were visualized by UV light (365 nm), a phosphomolybdic acid solution 10 wt. % in methanol or a vanillin solution (6 g of vanillin, 450 mL of ethanol, 40 mL of AcOH and 30 mL of H_2_SO_4_).

**Spectroscopic data**

**Compound 3:**

^1^H-RMN (500 MHz, CDCl_3_) δ: 7.84 (m, 6H), 7.78 (m, 3H), 7.68 (m, 6H), 6.00 (m, 1H), 5.28 (m, 1H), 4,77 (dd, 2H, *J*= 7.2, 14.7 Hz), 1.61 (t, 3H, *J*= 6.4 Hz)

^13^C-RMN (125 MHz, CDCl_3_) δ: 137.9 (CH, *J*=11.8 Hz), 134.9 (CH, *J*=3.0 Hz), 134.0 (CH, *J*=8.0 Hz), 130.3 (CH *J*=10.3 Hz), 118.3 (C, *J*=70.9 Hz), 114.8 (CH, *J*=8.2 Hz), 27.9 (CH_2_, *J*=40.7 Hz), 18.4 (CH_2_, *J*=2.3 Hz),

HR-ESI-MS m/z 317.1452; calculated 317.1459 for [C_22_H_22_P]^+^

**Compound 8:**

^1^H-RMN (500 MHz, CDCl_3_) δ: 7.89 (m, 6H), 7.78 (m, 3H), 7.70 (m, 6H), 3.79 (m, 2H), 1.61 (m, 4H), 1.22 (m, 6H), 0.80 (t, 3H, J= 7.0 Hz)

^13^C-RMN (125 MHz, CDCl_3_) δ: 134.9 (CH, *J*=3.0 Hz), 133.6 (CH, *J*=9.9 Hz), 130.5 (CH *J*=12.5 Hz), 118.4 (C, *J*=85.3 Hz), 31.2 (CH_2_), 30.3 (CH_2_, *J*=15.2 Hz), 28.9 (CH_2_), 22.8 (CH_2_, *J*=53.2 Hz), 22.6 (CH_2_), 22.4 (CH_2_), 13.9 (CH_3_),

HR-ESI-MS m/z 361.2087; calculated 361.2085 for [C_25_H_30_P]^+^

**Compound 13:**

C-13

^1^H-RMN (500 MHz, CDCl_3_) δ: 7.81 (m, 6H), 7.76 (m, 3H), 7.68 (m, 6H), 3.75 (m, 2H), 1.59 (m, 4H), 1.18 (m, 18H), 0.84 (t, 3H, J= 7.1 Hz)

^13^C-RMN (125 MHz, CDCl_3_) δ: 134.9 (CH, *J*=3.0 Hz), 133.7 (CH, *J*=9.8 Hz), 130.5 (CH *J*=12.3 Hz), 118.6 (C, *J*=85.3 Hz), 31.8 (CH_2_), 30.4 (CH_2_, *J*=15.1 Hz), 29.6 (2xCH_2_), 29.5 (CH_2_), 29.4 (CH_2_), 29.3 (CH_2_), 29.2 (CH_2_), 29.1 (CH_2_), 22.8 (CH_2_, *J*=45.6 Hz), 22.6 (2xCH_2_), 14.0 (CH_3_),

HR-ESI-MS m/z 445.3013; calculated 445.3024 for [C_31_H_42_ P]^+^

**Compound 17:**

^1^H-RMN (500 MHz, CDCl_3_) δ: 7.85 (m, 6H), 7.77 (m, 3H), 7.68 (m, 6H), 3.84 (m, 2H), 1.63 (m, 4H), 1.19 (m, 28H), 0.84 (t, 3H, J= 7.0 Hz)

^13^C-RMN (125 MHz, CDCl_3_) δ: 134.9 (CH, *J*=3.0 Hz), 133.7 (CH, *J*=9.8 Hz), 130.4 (CH *J*=12.3 Hz), 118.6 (C, *J*=85.3 Hz), 31.9 (CH_2_), 30.4 (CH_2_, *J*=15.1 Hz), 29.7 (5xCH_2_), 29.6 (CH_2_), 29.5 (CH_2_), 29.4 (CH_2_), 29.3 (CH_2_), 29.2 (CH_2_), 29.1 (CH_2_), 22.7 (CH_2_, *J*=45.6 Hz), 22.5 (3xCH_2_), 14.0 (CH_3_),

HR-ESI-MS m/z 515.3810; calculated 515.3807 for [C_36_H_52_P]^+^

**Compound 18:**

^1^H-RMN (500 MHz, CDCl_3_) δ: 7.85 (m, 6H), 7.78 (m, 3H), 7.69 (m, 6H), 3.84 (m, 2H), 1.60 (m, 4H), 1.20 (m, 30H), 0.86 (t, 3H, *J*= 7.0 Hz)

^13^C-RMN (125 MHz, CDCl_3_) δ: 135.1 (CH, *J*=3.0 Hz), 133.9 (CH, *J*=9.8 Hz), 130.6 (CH *J*=12.3 Hz), 118.6 (C, *J*=85.3 Hz), 32.1 (CH_2_), 30.1 (CH_2_, *J*=15.1 Hz), 29.9 (6xCH_2_), 29.8 (CH_2_), 29.7(2xCH_2_), 29.6 (CH_2_), 29.5 (2xCH_2_), 29.4 (2xCH_2_), 29.1 (CH_2_), 22.9 (CH_2_, *J*=45.6 Hz), 14.3 (CH_3_)

HR-ESI-MS m/z 529.3964; calculated 529.3963 for [C_37_H_54_P]^+^

**Compound 19:**

^1^H-RMN (500 MHz, CDCl_3_) δ: 7.78 (m, 9H), 7.69 (m, 6H), 3.64 (m, 2H), 3.34 (m, 2H), 1.58 (m, 6H), 1.21 (m, 10H)

^13^C-RMN (125 MHz, CDCl_3_) δ: 176.9 (COOH), 135.0 (CH, *J*=3.0 Hz), 133.7 (CH, *J*=9.9 Hz), 130.5 (CH *J*=12.2 Hz), 118.3 (C, *J*=85.3 Hz), 33.3 (CH_2_), 30.2 (CH_2_, *J*=15.4 Hz), 28.8 (CH_2_), 28.71 (CH_2_), 28.69 (CH_2_), 28.64 (CH_2_), 28.62 (CH_2_), 24.7 (CH_2_), 22.7 (CH_2_, *J*=44.5 Hz), 22.5 (CH_2_)

HR-ESI-MS m/z 447.2458; calculated 447.2453 for [C_29_H_36_O_2_P]^+^

**Compound 20:**

^1^H-RMN (500 MHz, CDCl_3_) δ: 7.83 (m, 18H), 7.73 (m, 12H), 3.74 (m, 4H), 1.71 (m, 8H), 1.62 (m, 6), 1.17 (m, 6H),

^13^C-RMN (125 MHz, CDCl_3_) δ: 135.0 (CH, *J*=3.0 Hz), 133.6 (CH, *J*=9.9 Hz), 130.5 (CH *J*=12.5 Hz), 118.4 (C, *J*=85.3 Hz), 30.2 (CH_2_, *J*=15.2 Hz), 29.0 (CH_2_), 28.9 (CH_2_), 28.8 (CH_2_), 22.9 (CH_2_, *J*=53.2 Hz), 22.5 (CH_2_),

EIMS m/z 346 [C_48_H_54_P_2_]^2+^
